# Mycelial growth of wood fungus *Ganoderma sessile* in porous scaffolds

**DOI:** 10.1016/j.mtbio.2025.102282

**Published:** 2025-09-10

**Authors:** Natalie Nussbaum, Nils Repond, Antoni Gandia, Peter Fischer, Patrick A. Rühs

**Affiliations:** a Eidgenössische Technische Hochschule Zürich, Department of Health Sciences and Technology, 8092 Zürich, Switzerland; b Eidgenössische Technische Hochschule Zürich, Department of Materials, 8093 Zürich, Switzerland; c Polytechnic University of Valencia, Institute for Plant Molecular and Cell Biology, 46022 Valencia, Spain

**Keywords:** Mycelium, Foam, Scaffold, Host material, Ergosterol, Biomass, Engineered living materials

## Abstract

The mycelium of filamentous wood fungi exhibits adaptive growth strategies influenced by their host material. In 2D solid-state fermentation, densely packed substrates limit oxygen access, resulting in hyphal growth mainly at the substrate-air interface. To address this challenge, we investigated open porous scaffolds as growth environments for the filamentous fungus *Ganoderma sessile*, quantifying mycelial biomass formation via ergosterol content. This quantitative approach directly demonstrates a scaffold-associated biomass increase of 60% after 7 days compared to plate cultures and thus provides experimental evidence linking increased accessible surface area in porous substrates to enhanced biomass formation in solid-state fungal growth. Mycelium colonization of the hydrogel scaffold also enhanced their mechanical properties, including stiffness and elastic recovery. This scaffold-associated biomass increase was not observed for *G. lucidum* or *P. ostreatus*, underscoring a species-specific effect with ergosterol levels for *G.sessile* peaking at 2 wt% malt extract in the substrate, in contrast to both higher and lower malt extract concentrations. These findings improve our understanding of solid-state fermentation, highlight the importance of species-specific responses and guide the design of substrates for fungi-based materials with tailored properties.

## Graphical abstract

**Figure:**
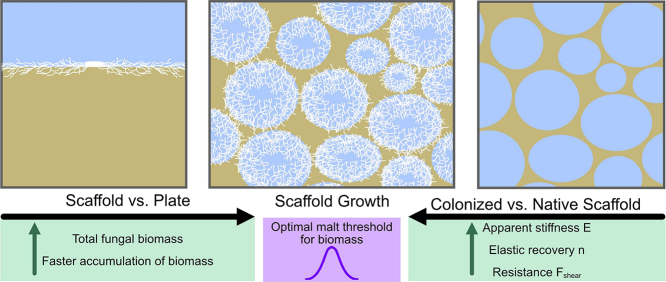

## 1 Introduction

Mycelium, the vegetative growth form of filamentous fungi, is a material of remarkable properties, such as high mechanical strength [1] and self-healing potential [2], [3]. Moreover, the mycelium’s ability to adjust its growth to different host materials makes it highly suitable for creating engineered living materials, where microorganisms respond to environmental cues [3], [4], [5], [6]. In addition, mycelium is biocompatible and has gained popularity as a sustainable material in a variety of industries; from packaging [7], [8] to innovative construction materials [9], [10], where it is commonly used to bind and reinforce discrete particles, forming composites. Mycelial biomass can be grown via liquid-state fermentation (LSF) or solid substrates through solid-state fermentation (SSF). SSF plays a crucial role in applications such as packaging materials or food products, where mycelium proliferates on solid-like substrates and within three-dimensional structures. However, growth on solid substrates is limited by surface availability and oxygen diffusion, and if the substrate is densely packed, mycelial growth occurs mainly at the substrate surface.

The proliferation of filamentous fungi in solid-state fermentation (SSF) is influenced by several key factors, including water availability [11], nutrient abundance [12], host material properties [6], [13], [14] and oxygen diffusion [15], [16]. An adequate moisture content is essential to support fungal metabolism [11], while a sufficient supply of nutrients is necessary for sustained growth [12]. Host material hardness can influence the density of mycelial network [13] and its penetration into the material [14]. Oxygen supply, often limited in dense or compacted substrates, is crucial in maintaining aerobic conditions and enabling efficient biomass production [15], [16]. These factors must be carefully balanced to optimize fungal growth and metabolic activity in SSF systems. As mycelium grows on solid material, the hyphae penetrate the substrate matrix but mainly develop at the solid-air interface, where they can absorb oxygen [17]. The significance of oxygen in SSF systems is widely recognized, and models are employed to enhance oxygen transfer in bioreactors for mycelium biomass production [18], [19]. Facilitating mycelial growth throughout a solid substrate can be achieved by employing porous materials that improve oxygen diffusion. At the same time, increasing the interface area for mycelial growth presents a promising strategy to enhance fungal biomass by providing more surfaces for hyphal attachment. However, it is essential to ensure that other growth-limiting factors, such as water and nutrient availability near the hyphal network, are not restricted in the process.

One approach to addressing this challenge would be, for example, the development of open porous systems for mycelial proliferation. Hydrogel foams represent a distinct category of macroporous materials characterized by a dispersed gas phase within a continuous hydrogel phase [20]. They are widely used in the biomedical sector as scaffolds for cell culture and tissue engineering [21], [22]. Understanding how to promote mycelial biomass formation within a porous system is crucial for industrial applications including sustainable leather-like tissue manufacturing, fermented foods and mycoprotein production, and novel materials for packaging, reinforcement, and biosensors. In fungal leather production, optimizing mycelial growth can yield stronger and more durable materials with desirable attributes such as flexibility, texture, and thickness [23], [24]. In the development of fermented food products and mycoprotein, controlled mycelial growth on a food matrix can improve the texture, flavor, and nutritional profile of the final products [25], [26]. In construction, packaging and development of fungal biosensors, the novel materials produced by cultivating mycelium on these porous scaffolds could allow lightweight materials to adopt more complex 3D and 4D architectures and withstand significant stress [2], [27], [28], [29], [30], [31]. However, the isolated impact of a porous system on the growth of filamentous fungi has not been investigated. Understanding this relationship is a crucial step toward optimizing fungal-based materials for applications where scaffold architecture plays a key role in shaping growth dynamics, texture, and overall material performance.

We fabricate macroporous hydrogel foams using sterile pressure foaming as a host material for mycelial growth and compare these conditions to the widely used agar plate method for cultivating the filamentous fungus *Ganoderma sessile*. This comparison provides a valuable reference to highlight the effects of the 3D hydrogel environment on fungal growth. By increasing the water-air interface of the substrate, we investigate how this environmental change affects the growth dynamics and overall biomass yield of the mycelium. The impact of the host material is analyzed optically through electron microscopy, mechanically through compression testing, and finally, by quantifying fungal biomass across the different substrates.

## 2 Materials and methods

### Materials

All materials and equipment used for fungal cultivation were prepared in a sterile environment in a SafeFAST Premium 215 bench (Faster, Italy). This research was conducted mainly using a single strain of the *Ganoderma sessile* species (col. code 95-19, MOGU, Italy), from the Basidiomycetes family. For one experiment, two additional strains from the same phylum were used: *Ganoderma lucidum* (col. code MG11500, Mycogenetics, Germany) and *Pleurotus ostreatus* (MG1005, Mycogenetics, Germany). All raw materials for the growth substrate were received as powders. Malt extract and agar were received from Morga AG (Switzerland) and yeast extract from Thermo Fisher Scientific (Germany). For the ergosterol extraction, HPLC-grade methanol (99.9%), HPLC-grade chloroform (99.9%), ergosterol standard (Certified Reference Material) and cholesterol standard (Certified Reference Material) were sourced from Sigma Aldrich (Germany).

### Methods

#### Maintenance of fungal cultures

Cultures were maintained in vented 90 mm-diameter petri dishes (VWR, USA), sealed with parafilm (Bemis Company Inc., USA). They were incubated in the dark at 30 °C on a defined standard malt agar substrate (SMA), containing 2 wt% malt extract (ME), 2 wt% agar, and 0.2 wt% yeast extract and transferred to a new plate weekly. Only the malt concentration in the SMA substrate was adjusted from 2 wt% to 0.5–4 wt% at one point in this study. All incubation events were run in the dark at 30 °C and 80% relative humidity. For all experiments, the inoculum was consistently taken from the edge of a max. 5-day-old radial fungal colony grown on agar to ensure the use of fresh and active culture material.

#### Hydrogel scaffold preparation

Foamed agar hydrogel scaffolds were obtained through a membrane foaming process. After autoclaving, the liquid substrate mixture was tempered at 47–48 °C before foaming. The selected temperature range was chosen to maintain the process just above the gelation temperature (46.26 °C, SI Figure S1) while avoiding excessive heat. This controlled temperature allowed the substrate to initiate gelation and stabilize the bubbles during the foaming process. A defined volume of liquid substrate mixture (27 ml) was poured into a sterile fritted Büchner funnel (porosity grade 3, Schott AG, Germany) connected to an air pressure controlling unit, allowing one to adapt the airflow coming at the lower part of the fritted disc. The foaming of the mixture was performed at a constant flow rate of 800 mL/min, to achieve mono-disperse foams [20], [32], and the mixture was left to expand into the funnel for 10 to 15 s. Finally, the foam was decanted into a 94 × 16 mm petri dish and left for solidification on top of a cooling element before hermetically sealing the lid with parafilm. Scaffolds for mycelial cultivation were prepared by point inoculation: A standardized size of inoculum (5 mm diameter) was placed in the middle of the scaffold sample. The samples were then sealed and incubated in the dark at 30 °C and 80% relative humidity.

#### Biomass quantification

Previous studies have demonstrated that ergosterol content provides a reliable linear approximation of fungal biomass [33], [34]. This method allows for quantitative comparisons of biomass across different substrates. To compare biomass formation on both the plate substrate and the scaffolds, the same volume of substrate was used. After cultivation, the mycelium samples were freeze-dried using a FreeZone 4.5L Benchtop freeze dryer (Labconco, USA) and then ground into homogenized powders. Ergosterol was extracted using a modified method based on Alekseyeva et al. [34] and described in detail in Nussbaum et al. [35]. This extraction process involved methanol and chloroform as solvents. To isolate the total lipid fraction, we applied the standardized lipid extraction method by Bligh and Dyer [36]. The total weight of the freeze-dried substrate-mycelium mix for each sample was recorded and 300 mg of this mix was weighed into 30 ml pyrex tubes. The tubes were wrapped in foil to protect them from UV rays and prevent potential degradation of ergosterol. To each sample, 1 ml of an internal standard solution (2 mg/ml cholesterol in 95:5 chloroform-methanol) and 9 ml of a 2:1 chloroform-methanol solvent were added. The samples were then vortexed and sonicated for 20 min at room temperature. Following this, 4 ml of milli-Q water was added to each sample, and the mixture was vortexed and sonicated for another 20 min. The samples were then centrifuged at 1000 g for 3 min using a 5810 R centrifuge (Eppendorf, Germany). After centrifugation, clear phase separation was observed. Sterols were extracted by transferring the lower phase into clean vials using a glass pipette. A second washing step was performed by adding 9 ml of chloroform to the original vials, mixing, centrifuging, and again transferring the lower phase to the clean vials. The solvent in the vials was evaporated under a nitrogen stream at 40 °C. After evaporation, 1 ml of a 95:5 chloroform-methanol solvent was added to redissolve the cholesterol and ergosterol. The samples were sonicated at room temperature for 20 min to ensure complete dissolution, which was verified optically. Finally, the samples were filtered through 0.45μm nylon filters and transferred to amber HPLC vials for subsequent analysis. The samples were analyzed using high-performance liquid chromatography (HPLC) with an Agilent 1200 series system (Agilent Technologies, USA) equipped with a Nucleodour C 18 ec column (Macherey-Nagel, Germany) and quantified using the Chromeleon software (Chromeleon 7.2.7, Thermo Scientific, USA). Cholesterol and ergosterol concentrations were determined by integrating the peaks [mAU/min] measured via HPLC at specific wavelengths: 205 nm for cholesterol [37], [38] and 282 nm for ergosterol [33], [34]. The ergosterol concentration in each sample was quantified by adjusting the measured values based on the recovery of the internal standard in each sample, as well as the total weight of the sample. The quality of the extraction was confirmed, with the internal standard recovery being over 87% for all samples.

### Scaffold characterization

The hydrogel scaffolds were characterized by different methods: Microtomography, material density, and mechanical analysis.

#### Microtomography

The internal structure of the foams was analyzed using a micro-CT 40 X-ray Microtomograph (SCANCO Medical AG, Switzerland). The samples were cut into cylindrical shapes with a diameter of 2.8 mm to fit the dimensions of the sample holder. The generated images were processed using the SCANCO application, with pore size analysis and 3D visualization performed within the software.

#### Scanning electron microscopy

The samples were initially fixed with vapor from glutaraldehyde, followed by osmium tetroxide vapor. After fixation, the samples were dehydrated using an ascending series of ethanol under vacuum conditions. Once incubated in dry ethanol, the samples were dried using critical point drying (Tousimis CPD 931, USA). Cross sections were then cut using fresh razor blades. The trimmed samples were mounted onto SEM stubs with conductive carbon cement and were subsequently sputter-coated with a 5 nm layer of Pt/Pd using a CCU-010 sputter coater (Safematic, Switzerland). Imaging was performed using either a Hitachi SU5000 (Japan) or TFS Magellan 400i Field Emission Scanning Electron Microscope (USA) at 3 kV by secondary electron detection.

#### Mechanical characterization

The samples underwent mechanical characterization through uniaxial compression tests, stress-relaxation tests, and Warner-Bratzler tests. Uniaxial compression and Warner-Bratzler tests were conducted using a Zwick testing machine equipped with a 10N measuring cell (ZwickRoell GmbH & Co. KG, Germany). Stress-relaxation tests were performed with an MCR 702 rheometer featuring a sand-blasted plate geometry PP25 (Anton Paar, Austria). For all mechanical tests, the scaffolds were cut into 20 mm cylinders to fit the measuring geometries, and a preload of 0.01 N was applied to ensure consistent conditions. In the uniaxial compression test, a piston geometry was used to compress the samples at a fixed speed of 1 mm/min. The effect of mycelial colonization on the stiffness of the samples was evaluated by comparing Young’s moduli, which were calculated as the ratio of stress to strain in the linear elastic region (45%–50%) of the compression curves (SI Figure S3). The quality of the linear fit was confirmed with all adjusted R-squared values exceeding 0.95.

For the stress-relaxation test, axial compression measurements were performed at a constant speed of 10 μm/s, followed by relaxation of the sample. Initially, the sample was compressed to a defined strain of 20%, after which it was allowed to relax for 300 s while the extensional force was recorded. Following this relaxation period, the sample was further compressed to a strain of 50% and subjected to a second relaxation phase for 300 s. This process was then repeated for a final strain of 70%, also with a corresponding relaxation period of 300 s.

In the Warner-Bratzler test, a blade geometry was used, cutting through the sample at a displacement speed of 50 mm/min. The shear force required to cut through the scaffold was measured.

### Descriptive statistics

To compare the mean value of ergosterol and Young’s modulus between two conditions, an independent two-sample t-test was performed. Prior to the analysis, the assumptions of normality and homogeneity of the variances were evaluated. If the variances were homogeneous, a standard independent two-sample t-test was performed. If there was a significant difference in variances, a Welch’s t-test was performed. To compare the ergosterol data across multiple fungal strains and conditions, we performed a two-way ANOVA and pair-wise comparisons to assess the effects of fungal strain and growth condition (scaffolds vs. plates) and their interaction. The significance level was established at p < 0.05. In the figures presented, asterisks (*) are used to indicate levels of statistical significance and p-values are reported as follows: p<0.05 (*), p<0.01 (**), p<0.001 (***) and p<0.0001 (****). Detailed results from the statistical tests are listed in Supplementary Information Tables S1-5. The statistical analysis was performed using Origin Pro 2021 9.8.0.200 software (OriginLab Corporation, Northampton, MA, USA).

## 3 Results and discussion

**Fig. 1:**
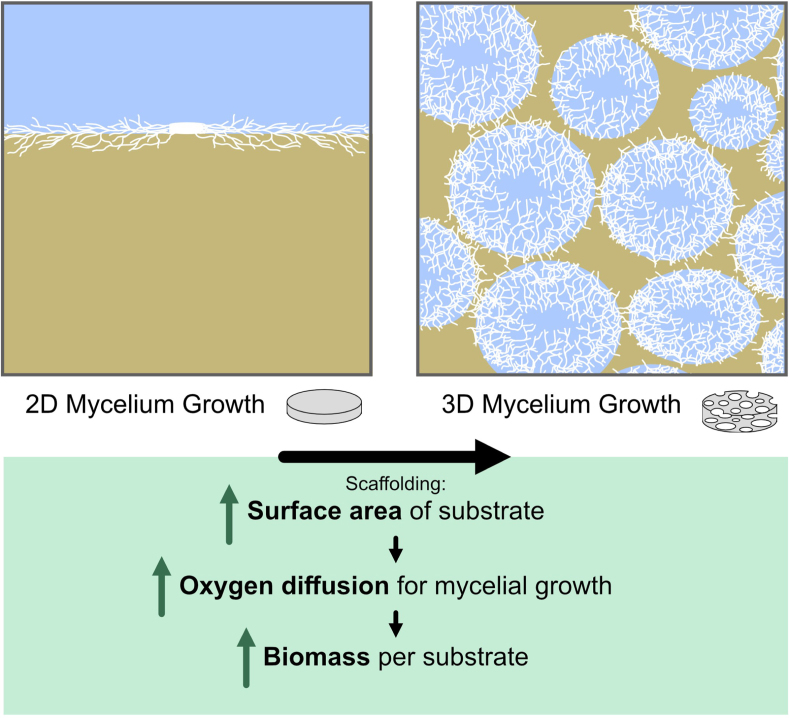
Schematic illustration of the potential impact of scaffolding in solid-state fermentation. Enhanced surface area in the scaffold substrate promotes improved oxygen diffusion. This is hypothesized to result in greater fungal biomass production in a 3D scaffold substrate compared to a 2D plate substrate.

To increase the surface area of the solid substrate, we employed pressure foaming, which resulted in a larger surface area and improved oxygen diffusion throughout the host material. This design promotes 3D mycelium growth across the substrate. In contrast, using a 2D plate substrate limits mycelial growth due to the dense packing of the material, which restricts oxygen diffusion. We hypothesize that this approach will result in an increased mycelial biomass per substrate within the scaffold. This concept is illustrated schematically in Fig. 1. A schematic of the workflow for all experiments is provided in SI Figure S4.

### 3.1 Native scaffold characterization

**Fig. 2:**
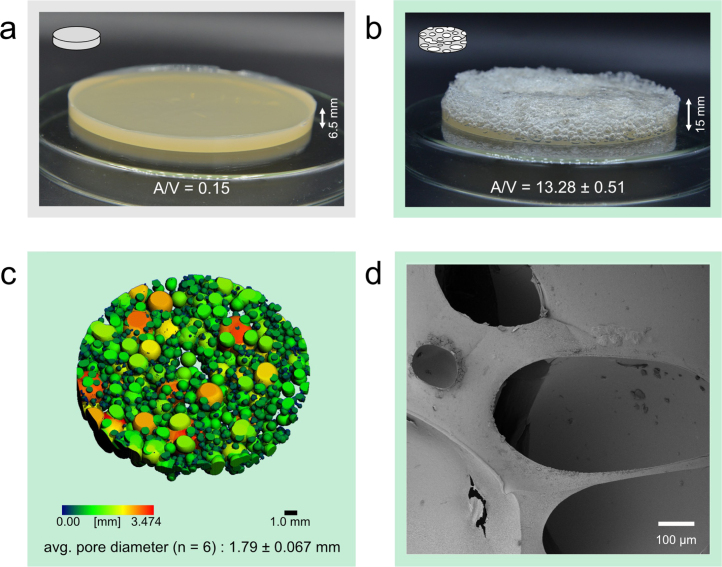
Photographs of the hydrogel plate (a) scaffold (b), and calculated surface area-to-volume (A/V) ratios. (c) Example micro-CT result illustrating pore size distribution and mean pore diameter (n=6) of hydrogel scaffolds. (d) Representative SEM image of the hydrogel scaffold.

The properties of the scaffolds were evaluated using optical imaging with a camera, non-destructive structural analysis via micro-computed tomography (micro-CT), and physical characterization through compression analysis and density measurement. Fig. 2 displays photographic representations of (a) a standard agar plate and (b) a foamed agar scaffold. With the same substrate volume and mold area (petri dish), the agar plate results in a height of 6.5 mm, while foaming results in a scaffold with 15 mm height, yielding an overrun of 130.77%. Considering the total volume and the surface area of an agar plate and scaffold (based on micro-CT data) the surface-to-volume ratio A/V increases from 0.15 mm^2^/mm^3^ to 13.28 mm^2^/mm^3^ (SE 0.51) for the foamed scaffold.

Fig. 2 c shows a representative example of the micro-CT output of a foamed scaffold, including the calculated average pore diameter (n = 6). Please refer to the Supporting Information Figure S2 for micro-CT images from all replicates. The pore size distribution is relatively heterogeneous, which is also reflected in the SEM image of the native scaffold (Fig. 2 d). The manual steps involved in pouring the formed foam into petri dishes did not allow for precise control over the homogeneity of the scaffold design. Given the need to conduct the foaming process in a sterile laboratory workbench environment, achieving a perfectly homogeneous pore size distribution was not a primary goal; therefore, the observed variability was considered acceptable. It is important to note that variations in pore size distribution can impact fungal growth by creating microenvironments that affect nutrient availability and gas exchange [2], [39]. Differences in pore size can also impact mycelium’s adhesion and the speed at which it can bridge air gaps [2]. For future studies, understanding these effects will be crucial for optimizing scaffold design, as controlled pore sizes could enable systematic investigation of how pore distribution influences nutrient availability, gas exchange, and fungal colonization. However, our research focused primarily on the effects of an overall increased surface area of the host material, rather than on achieving a controlled, uniform pore size distribution. Despite this, the produced scaffolds demonstrated reproducibility in their heterogeneity, as indicated by a small standard error (SE = 0.067 mm) of the mean pore size (1.79 mm) across six replicates (Fig. 2 c).

### 3.2 Fungal growth within three-dimensional scaffold

**Fig. 3:**
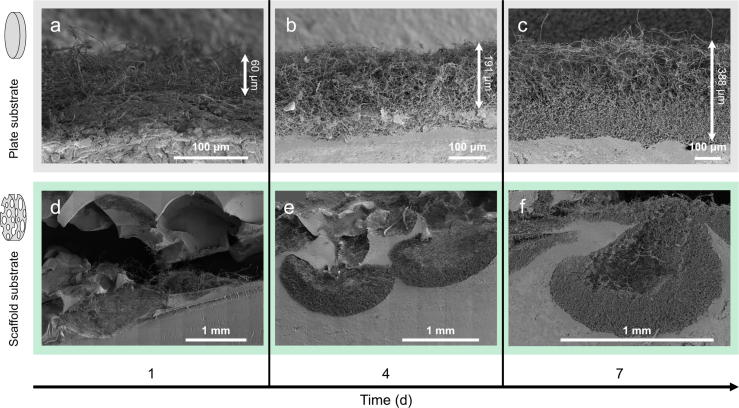
SEM images depicting mycelial growth on SMA substrate in plates (a-c) and foam substrate (d-f) after 1, 4, and 7 days of incubation. Arrows indicate the height of mycelial mats on the plates, with numbers representing a qualitative estimate of mat height.

Fungal growth on scaffold and control agar plates was assessed qualitatively using SEM imaging and quantitatively by determining the fungal biomass produced, as well as by assessing the stiffness and elastic recovery of overgrown scaffolds. To ensure a fair comparison between plate and scaffold growth, equal amounts of substrate were used for all samples. Fig. 3 displays representative SEM images of mycelium growth over time, captured at the same distance from the inoculum. In the control plates, the thickness of the mycelium mat increases over time, as indicated by white arrows in Fig. 3 a-c. In the scaffold samples, while the pores were scarcely occupied after 1 day, colonization became denser after 4 days, and by day 7, most pores were filled with mycelium (Fig. 3 d-f). These images also indicate that mycelium growth predominantly occurs at the substrate-air interface, with minimal hyphal penetration into the substrate.

Three factors were considered to compare the accumulated biomass: The increase in kinetics of biomass, the effect of malt concentration in the substrate on biomass, and the biological variability among different fungal strains.

#### 3.2.1 Kinetics of biomass accumulation

**Fig. 4:**
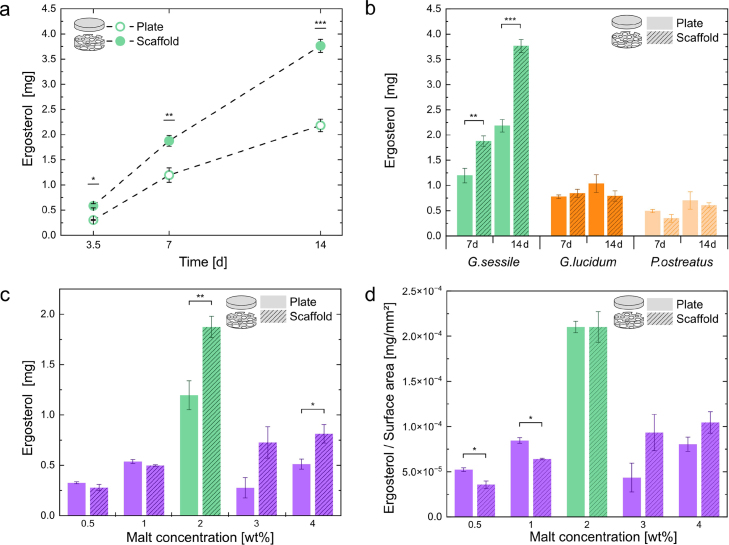
(a) Ergosterol measured over time of *G.sessile* grown on plate and scaffold substrate. b) Ergosterol measured after 7 and 14 days for various Basidiomycota strains grown on plate and scaffold substrate. c) Ergosterol of *G.sessile* grown on plate and scaffold samples with varying malt concentrations after 7 days. d) Ergosterol data from (c) normalized to the surface area of plate and scaffold samples. Error bars represent the standard error of the mean (SEM), n = 4 for all ergosterol data.

At all time points measured, biomass accumulation was significantly greater in scaffold growth compared to plate growth for *G.sessile*. Moreover, biomass accumulated more rapidly in scaffolds than in plates (Fig. 4 a). The biomass of *G. sessile* grown on both scaffold and plate substrates was quantified at various incubation intervals. The values are presented as ergosterol quantity in a sample, where the growth substrate quantity is consistent across samples.

Ergosterol quantification is a well-established method for studying fungal biomass, as ergosterol concentration is linearly related to fungal biomass [33], [34]. In this context, the increase in oxygen diffusivity and the expanded interface area enhance the growth vigor of *G.sessile*. The faster increase in biomass accumulation in scaffold growth can be attributed to two factors. First, the three-dimensional nature of the scaffold provides a larger surface area that supports enhanced mycelial growth by offering more attachment points for fungal hyphae [17]. Second, the macro-porosity of the scaffold improves oxygen transfer, which supports increased fungal proliferation [40].

#### 3.2.2 Biological variability in biomass yield

The biological variability among different fungal strains has a strong influence on the biomass yield across various substrates. In addition to *Ganoderma sessile*, two other fungal strains were studied: *Ganoderma lucidum*, which is from the same genus, and *Pleurotus ostreatus*, a Basidiomycota that is not closely related. Fig. 4 b presents the results of the ergosterol quantification of these three strains after 7 and 14 days of incubation. The biomass yield was highest for *G. sessile* compared to the other strains. Previous research has indicated that *G. sessile* produces higher biomass yields than other *Ganoderma* strains [41].

For both incubation periods, only *G. sessile* showed a significant increase in growth when cultivated on scaffold substrates compared to plate substrates. Additionally, we observed an overall increase in biomass formation over time, except for *G. lucidum* grown in scaffolds. Results from pair-wise comparisons of ergosterol data after 7 days, show that no significant effect on ergosterol of the scaffold substrate was found for *G.lucidum* and *P.ostreatus*. Further, ergosterol data was significantly higher for *G.sessile* compared to *G.lucidum* and *P.ostreatus* both on plate and foam scaffolds (Detailed results in SI Table S1-2).

Compared to *G.lucidum* and *P.ostreatus*, *G.sessile* exhibits faster radial growth rates on agar plates, as reported in our previous work [12]. These documented growth rate differences provide quantitative support for our hypothesis that the lower biomass production of *G.lucidum* and *P.ostreatus* in scaffold substrates is linked to their inherently slower growth dynamics compared to *G.sessile*.

We hypothesize that the effect of scaffold growth is either reduced or not observed for *G.lucidum* and *P.ostreatus* due to their slower growth rates. This slower growth likely prevents them from benefiting from advantages such as improved oxygen diffusion and increased surface area within the same timeframe as *G.sessile*. Furthermore, successful colonization of scaffolds depends on the capacity of hyphae to bridge air gaps and connect nutrient-rich zones; strains with slower growth or less extensive branching may be less able to take advantage of these scaffold properties. The observation that biomass levels plateau for *G.lucidum* after 7 days, while *G.sessile* and *P.ostreatus* continue to accumulate biomass, suggests that the scaffold material may not provide optimal support for *G.lucidum*, whereas other strains may respond differently depending on their growth dynamics and hyphal morphology.

#### 3.2.3 Effect of malt concentration on biomass yield

After a 7-day incubation period of *G.sessile* on the standard substrate, the scaffold growth yielded significantly more biomass than the plate growth (Fig. 4 c). In this experiment, mycelium was grown on a standard substrate consisting of 2 wt% malt extract (ME), 2 wt% agar, and 0.2 wt% yeast extract, a formulation that has previously been shown to support optimal mycelial growth kinetics for *G.sessile* [12].

When using substrates with 0.5 and 1 wt% ME, no significant difference was observed between scaffold and plate growth. This finding is also evident in the photographs depicting the morphology of the cultures (SI Figure S5), where the mycelium appears more sparse on both the plates and scaffolds with 0.5 and 1 wt% substrates.

The mycelium mat on the plate substrate appears more dense with higher ME concentrations of 3 and 4 wt%. In addition, mycelium cultivated on 3 and 4 wt% ME substrates resulted in an increased biomass formation in the scaffold compared to the plate substrate, as previously seen with the 2 wt% ME substrate. The relationship between the quantified ergosterol and ME concentration reveals a pattern that suggests an optimal ME concentration for achieving maximal ergosterol levels. Beyond this point, increasing the ME concentration does not lead to a higher accumulation of ergosterol within the given time frame

The anticipated increase in biomass concentration with higher ME concentrations in the lower ME concentration regime of 0.5–2 wt% can be attributed to the increasing abundance of available nutrients, encouraging mycelial proliferation, and, consequently, higher ergosterol levels. The water activity was approximately 0.99 across all substrates (SI Figure S6). Thus, the observed differences in biomass are more likely due to the presence/absence of ME, rather than variations in water activity within the substrates. While we cannot completely exclude subtle effects from nutrient imbalances or metabolic regulation at higher ME concentrations, the fact that ergosterol accumulation peaks at 2 wt% ME suggests that these factors are unlikely to be the primary cause of the observed growth patterns. The production of biomass in fungal systems during solid-state fermentation (SSF) is largely dependent on the number of hyphal tips and the rate of their extension, both of which are closely linked to glucose affinity. In simpler terms, when sugars are more readily available, there is an increase in tip formation and extension, resulting in greater biomass production [42]. Specifically, previous studies have shown that higher concentrations of sugars in the solid substrate lead to increased metabolite production in filamentous fungi [43], [44]. This finding is consistent with earlier research on *G.sessile*, which found that higher malt concentrations in the substrate resulted in increased stiffness and network density [12].

However, there appears to be a threshold for ME concentration in the solid substrate, beyond which adding more ME does not result in increased biomass formation. This is consistent with our previous study, where a 5 wt% ME substrate did not significantly improve the growth kinetics of *G.sessile* and appeared to slow radial growth compared to the 2 wt% ME substrate [12]. Similarly, Gantenbein et al. [2] observed that low concentrations of ME promote the radial expansion of fungal mycelium, although their research focused on higher concentration ranges (2–20 wt%).

Herein, the observed lower ergosterol at ME concentrations of 3 and 4 wt% likely arise from exploitation and exploration strategies that *G. sessile* exhibits. They may find a balance between slower hyphal extension but denser network formation [2], [5], [12], [45], [46]. By growing in a denser network, *G.sessile* may benefit more from enhanced oxygen exchange, ultimately supporting more efficient metabolic activity and leading to higher overall biomass production during scaffold growth.

Thus, we interpret the observed increase in ergosterol levels up to 2 wt% ME as a reflection of enhanced metabolic activity, consistent with previous literature [43], [44]. The subsequent decrease in ergosterol at 3 to 4 wt% ME can be explained using the “exploitation-exploration” strategy, previously demonstrated at similar malt extract concentrations [2], [12]. Nonetheless, further studies, such as real-time single hypha observations, could provide deeper insights into the cellular mechanisms driving scaffold colonization.

To investigate this effect, we recalculated the biomass accumulated per surface area of the substrate for both plate and scaffold. Significantly higher ergosterol levels per surface area for plate growth were found for 0.5 and 1 wt% ME substrate (Fig. 4 c). We assume that the quantified ergosterol per surface area in these cases is lower for scaffold growth due to the scaffolds’ higher estimated surface area of the scaffold compared to the plate: The surface area of the plate is 6.4 × 10${}^{3}\;{\mathrm{mm}}^{2}$, while the scaffold has an estimated surface area of 7.8 × 10^3^ mm${}^{2}$ (for detailed calculation, see SI Table S4). For ME concentrations ranging from 2 to 4 wt%, there is no significant difference between plate and substrate if you consider the biomass accumulated per surface area of the substrate.

In conclusion, the biomass/surface area metric does not capture the differences of mycelium growth in substrates of different macro-architectures beyond the surface area (i.e. thickness of agar bridges, pore distribution). The biomass, however, directly reflects that cumulative growth, revealing global differences in biomass hidden by the per-unit-area normalization. Despite an increase in ergosterol per unit surface area in plate growth, scaffold substrates support a greater overall biomass relative to the total nutrients available.

In summary, while this study focused on malt extract to maintain a manageable scope, future work exploring alternative nutrient sources and time intervals could provide further insights into biomass formation and scaffold colonization. Verification of the observed trends with other sugars, combined with more detailed analyses of gene expression and metabolic pathways, would help clarify how nutrient availability influences mycelial growth and scaffold interaction. Such studies would strengthen our understanding of the mechanisms underlying species-specific responses and scaffold colonization dynamics.

### 3.3 Mechanical analysis of scaffolds

The mechanical properties of both native and colonized scaffolds were compared to evaluate the impact of mycelial colonization. We conducted three mechanical tests to understand how mycelial growth affects physical properties. First, we performed compression testing to assess how the proliferation of mycelium influences the stiffness of the composite material. Second, we carried out relaxation testing to observe the material’s behavior after the load was removed. Lastly, we tested the force needed to cut through the samples, which provided information about the internal structure and mechanical integrity of the scaffolds. These tests were designed to provide a comprehensive understanding of how mycelial colonization affects the mechanical performance of the scaffolds in various contexts, offering valuable insights for the application of mycelium-based biocomposite materials.

**Fig. 5:**
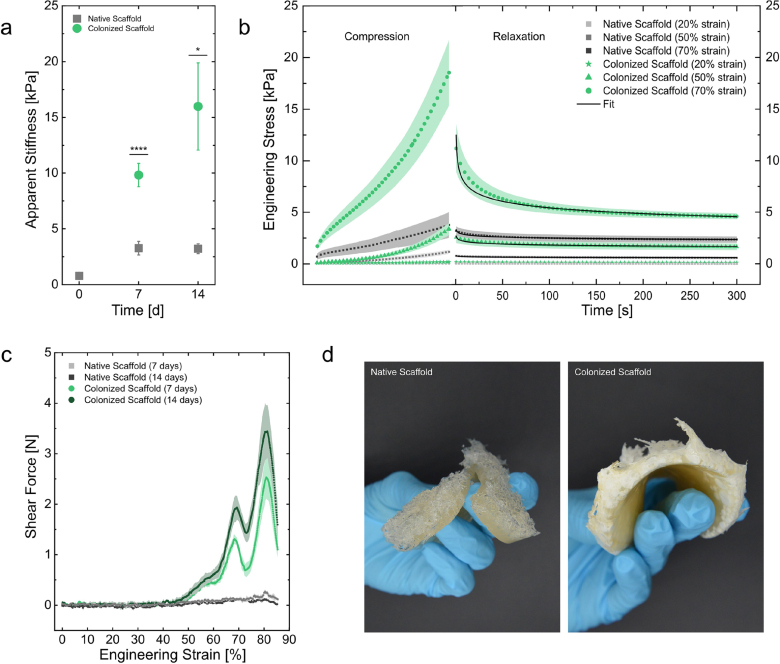
(a) Apparent stiffness (Young’s moduli) of native and colonized scaffolds after 7 and 14-day incubation periods. (b) Stress relaxation dynamics of native and colonized scaffolds (7-day incubation) following compression to various strain levels. (c) Force levels from a Warner-Bratzler test of native and colonized scaffolds after 7 and 14-day incubation periods during compression. (d) Brittleness and elasticity of native and colonized scaffolds (7-day incubation) by a finger bend test. Error bars and shaded bands indicate the standard error of the mean (SEM). n = 6 for Young’s moduli, n = 3 for Stress relaxation tests and for Warner-Bratzler test data.

The values for mean apparent stiffness (Young’s modulus) demonstrated a significant effect of *G.sessile* mycelial colonization when compared to the native scaffolds (Fig. 5 a). The native scaffold has an average stiffness of 0.79 kPa (SE 0.06 kPa), whereas the colonized scaffold showed an average stiffness of 9.82 kPa (SE 1.06 kPa) after 7 days of growth and 16 kPa (SE 3.90) after 14 days of growth. Thus, a significant increase in stiffness due to mycelium proliferation and subsequent reinforcement of the scaffold can be observed, both after 7 and 14 days of incubation.

The increased Young’s modulus in colonized scaffolds results from the interconnected mycelium network responding to axial forces. When the system is compressed, the interconnected mycelial network enhances its resistance, implying that the composite material’s elastic modulus is determined by the load-bearing strength of the mycelial network formed within the porous structure. The additional increase in resistance observed after 14 days of incubation can be attributed to the formation of more connection points within the network, or a general increased mycelial biomass formation (Fig. 4 a). Comparable findings were reported by Gantenbein et al. [2], showing values of apparent stiffness of overgrown printed scaffolds in a similar range (≈ 20 kPa after 14 days of growth) and a consistent increase in scaffold stiffness over time, which reflected the growth of the mycelium network within. Additionally, a study by Soh et al. [39] revealed that the stiffness of wood-based biocomposite materials could be enhanced by increasing porosity, thereby promoting the formation of dense mycelium networks. Similar results, indicating an increase in material stiffness with mycelial colonization, have been reported in literature [47], [48], [49].

While the apparent stiffness values measured here are much lower than those reported for mycelium-based materials in the literature (typically in the single-digit MPa range) [24], [50], our focus was on the relative increase in stiffness resulting from mycelial colonization compared to native scaffolds. Moreover, our samples were not prepared under the standardized conditions commonly used for myco-material characterization — such as specific drying protocols — so direct comparison of absolute values is not appropriate.

Given the observed reinforcement effects in axial compression, further research was conducted to examine the relaxation behavior of the scaffolds (Fig. 5 b). This approach provided deeper insight into the material response after load removal. Examining the compression curve up to 20% deformation, no distinct differences were observed between the native and colonized scaffold. At this low strain level, pore compression dominates, diminishing the effect of the mycelium, while the hydrogel fraction is more prominent. However, at higher strain levels of 50% and 70%, steeper slopes and higher maximum stress values are observed in the colonized scaffold compared to the native scaffold. The colonized scaffold exhibits greater stiffness, and this effect becomes more pronounced at higher strain levels. Further densification of the scaffold, with fewer compressible pores and an increased proportion of scaffold and mycelium material, amplifies the influence of the mycelial network on its mechanical properties.

After applying a 20% strain, the relaxation curves for both native and colonized scaffolds exhibit a similar shape, indicating that they respond to relaxation in the same way. In this context, we anticipate that the relaxation process is primarily influenced by the compression of air, with minimal impact from the material’s integrity. To compare the relaxation time at different strain levels of 50 and 70%, relaxation curves were fitted. The relaxation could be generalized using a power law:

(1) $$\sigma =k*t^{-n}$$

with using individual initial stress values for k as scaling factors and allowing the comparison of a relaxation rate n, defined as the negative exponent of time t. The parameter n indicates the rate at which the extensional stress σ decreases over time after compression. In scaffold relaxation after compression to 50% strain, the relaxation rate n is higher for the colonized scaffold (0.074, SE 1.77 10^−4^) than for the native scaffold (0.042, SE 1.96 10^−4^). This suggests that, for colonized scaffolds, the relaxation time is shorter, indicating that the material behaves more elastically. At 70% strain, this effect is more pronounced, with n being 0.16 (SE = 1.22 10^−4^) for the colonized scaffold and 0.053 (SE = 8.18 10^−5^) for the native scaffold.

Despite colonization of *G.sessile* reinforcing the scaffold, the mycelium itself is more flexible and porous than the hydrogel matrix. The mycelium network, particularly under high strain, can undergo internal rearrangement, such as the bending of hyphae, which helps in rapidly dissipating stress, leading to shorter relaxation times. In contrast, the native scaffold might relax more slowly due to the lack of such internal mechanisms. Hydrogels, which are viscoelastic materials, typically exhibit slow relaxation behavior as the polymer chains in the hydrogel take longer to return to their equilibrium positions. Without the additional connective points provided by the mycelium for stress relief, the hydrogel foam takes longer to dissipate stress, resulting in slower relaxation.

In compression-relaxation tests, the relaxation time is governed by the viscoelastic properties of the material. Generally, an increase in polymer density within a hydrogel leads to a longer relaxation time [51]. Another reason for the faster relaxation of colonized scaffolds may be the enzymatic degradation of nutrients into smaller molecules. This degradation can disrupt the polymer network, making it slightly more disordered and reducing the cross-link density of the polymer structure, which in turn results in faster relaxation. Additionally, the degradation of the agar substrate by mycelium releases water, which increases the plasticity of the hydrogel and enhances the flexibility of the polymer network.

To gain a better understanding of the internal structure of the scaffolds, Warner-Bratzler cutting tests were performed. Examining the force dynamics during these tests reveals distinct differences between the native and colonized scaffolds (Fig. 5 c). While the native scaffold exhibits a gradual increase in force throughout compression, the colonized scaffolds display two distinct force peaks: the first at 68% and the second at 80% strain. Notably, these peaks consistently occur at the same displacements across all replicates measured. The gradual increase in force observed during the compression of the native scaffold suggests a more uniform internal structure that offers consistent resistance to deformation. In contrast, the two force peaks seen in the colonized scaffolds suggest structural heterogeneity or distinct phases of resistance or rupture. These peaks likely correspond to mechanical responses related to the mycelial network or specific material layers being compressed. The first peak reflects the compression of the mycelium network within the scaffold, while the second peak may suggest the densification of multiple mycelial structures as the scaffold undergoes further compression, resulting in an increased number of connection points. Further, the appearance of the same force peaks at consistent displacements across all fungal foam replicates and after different times of incubation indicates that the structural properties of the fungal foam are highly reproducible, suggesting that the mycelial network and its interactions with the substrate are formed consistently during the growth process, leading to uniform mechanical properties.

These results confirm our earlier findings of increased stiffness due to *G.sessile* mycelial proliferation, as the measured compressive force is higher compared to the native scaffold. Furthermore, the presence of the two distinct force peaks indicates that mycelial growth occurs heterogeneously throughout the scaffold, rather than being confined to a single layer. We also observe an increase in resistance in the samples incubated for 14 days, which supports the findings shown in Fig. 5 a, suggesting that longer incubation periods correlate with higher stiffness measurements. The increased stiffness of the colonized scaffold is also visually demonstrated in a bending test, where both native and colonized scaffold samples were bent by hand. The native scaffold fractured almost immediately, while the colonized scaffold exhibited high elasticity, rupturing only in the thinner top layers (Fig. 5 d). While the mechanical characterization provides valuable insights into the physical properties of the scaffolds, future studies using confocal microscopy could help visualize mycelial growth patterns within the scaffolds. This would further clarify how the mycelium interacts with the scaffold’s pore structure and aid in interpreting the mechanical testing results.

## 4 Conclusion and outlook

**Fig. 6:**
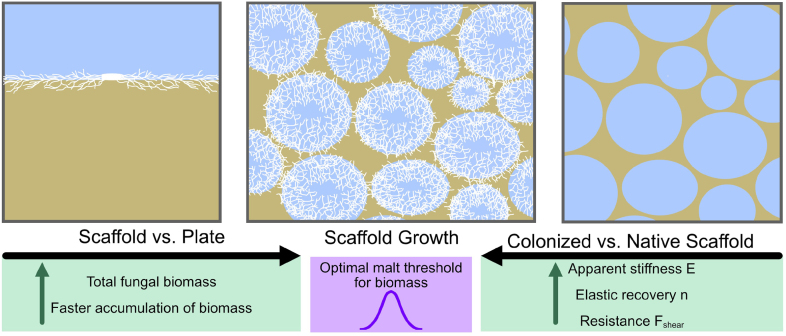
Schematic representation of the quantitative results from this study: Mycelium grown in scaffolds resulted in higher biomass and faster accumulation compared to standard plate growth (left box). An optimal malt concentration for maximal biomass accumulation was identified for *G. sessile* (middle box). Scaffolds colonized by fungal mycelium showed higher apparent stiffness, elastic recovery, and resistance in mechanical tests (right box).

We explored the preparation, cultivation, and evaluation of fungal mycelium growth on foamed hydrogel scaffolds. Sterile hydrogel foams were successfully produced and used as scaffolds for mycelial growth. SEM images demonstrated that the scaffold pores became increasingly filled with mycelium over time, while the mycelium mat in plate growth thickens progressively. Fig. 6 provides an overview of the key findings from quantitative analysis.

The ergosterol content increases with the incubation time in both plate and scaffold growth for *Ganoderma sessile*, with a faster increase observed in scaffold growth. This leads to a higher biomass in scaffolds compared to plate growth, which may be related to factors such as improved oxygen diffusion and a larger surface area for hyphal attachment compared to plate growth. However, this effect was not observed in the ergosterol accumulation of two other fungal strains *Ganoderma lucidum* and *Pleurotus ostreatus*. These results highlight that the growth-promoting effect of open porous scaffolds is species-specific and may depend on intrinsic physiological traits such as growth rate, hyphal density, and substrate utilization strategy. Recognizing such specificity is essential for the design of mycelium-based materials, as optimal scaffold parameters may need to be tailored to each fungal species to maximize performance.

While fungal biomass of *G.sessile* increased with higher malt extract concentration, the maximum ergosterol levels were observed at 2 wt% ME, indicating a complex relationship between ME concentration and biomass accumulation. The growth dynamics observed on plate and scaffold substrates at different malt extract concentrations can be attributed to the exploitation/exploration growth strategies employed by fungal mycelium. We hypothesize that these dynamics result from a balance between the immediate availability of nutrients and the need to explore new sources of nutrients in a mycelium network. Mycelium cultivated within a hydrogel scaffold further significantly alters the material’s properties, including stiffness and elastic recovery. These findings highlight the impact of host material on the growth of *G.sessile* during solid-state fermentation, emphasizing the role of an increased air-substrate interface in boosting biomass and mechanical properties. The hydrogel scaffold in this study serves primarily as a model system to investigate how fungal colonization enhances mechanical properties. Importantly, the approach is not limited to agar-based hydrogels—alternative scaffold materials, including food-grade matrices, could be used to create structured bio-composites. This highlights the potential of fungal growth to improve mechanical and structural properties in diverse applications, such as meat analogue production or other engineered living materials. In addition, this study focused on *G. sessile*, and future work could test additional species, nutrient sources, and scaffold pore architectures. Advanced imaging could also reveal 3D mycelial growth dynamics, guiding the design of optimized scaffolds for enhanced biomass and function.

We successfully cultivated filamentous fungal mycelium within a 3D porous scaffold, demonstrated the reinforcing capability of the mycelial network, and showed that both the macrostructure and the nutrient distribution of the host material have a significant impact on fungal biomass accumulation. By directly quantifying biomass via ergosterol content, we provide, to our knowledge, the first experimental evidence linking increased accessible surface area in porous scaffolds to enhanced biomass formation in solid-state fungal growth. These findings provide valuable information for the targeted design of host materials to optimize fungal proliferation. Despite the growing interest in fungal-based materials, limited research has explored how three-dimensional, macroporous scaffolds affect the growth behavior and biomass development of filamentous fungi. This study addresses this research gap through a species-specific comparison of mycelial growth in sterile macroporous hydrogel foams versus traditional agar-based cultivation, revealing that biomass enhancement occurs for *G. sessile* but not for *G. lucidum* or *P. ostreatus*. The insights gained here are relevant to emerging applications such as fungal leather, fermented food systems, and sustainable bio-composites, where the architecture of the host material impacts fungal growth behavior and final material properties.

## CRediT authorship contribution statement

**Natalie Nussbaum:** Writing – review & editing, Writing – original draft, Visualization, Investigation, Formal analysis, Data curation, Conceptualization. **Nils Repond:** Writing – original draft, Visualization, Investigation, Formal analysis. **Antoni Gandia:** Writing – original draft, Methodology, Investigation, Conceptualization. **Peter Fischer:** Writing – review & editing, Writing – original draft, Supervision, Resources, Project administration, Funding acquisition, Conceptualization. **Patrick A. Rühs:** Writing – review & editing, Writing – original draft, Supervision, Resources, Project administration, Funding acquisition, Conceptualization.

## Declaration of competing interest

The authors declare that they have no known competing financial interests or personal relationships that could have appeared to influence the work reported in this paper.

## Data Availability

Data will be made available on request.

## Appendix A Supplementary data

The following Supporting Information is available free of charge (PDF): Rheological determination of substrate gelation temperature in Figure S1; Micro-CT images of hydrogel scaffolds showing qualitative pore structure and distribution in Figure S2; Fitting of stress–strain curves for Young’s modulus calculation in Figure S3; Detailed results of statistical tests in Tables S1 - 5; Workflow schematic illustrating the experimental steps and corresponding analyses performed at each stage in this study in Figure S4; ;Morphological images of mycelial growth in Figure S5; Water activity values of substrate in Figure S6; Detailed calculations for surface areas in Table S6. The following is the Supplementary material related to this article.

MMC S1 Mycelium grown in porous scaffolds results in higher biomass and mechanical properties compared to standard plate growth.
