# Supplementary material for: Mycelial growth of wood fungus Ganoderma sessile in porous scaffolds
Source: Mater Today Bio. 2025 Sep 10;35:102282. doi: 10.1016/j.mtbio.2025.102282 (PMC12859639; doi:10.1016/j.mtbio.2025.102282)
Supplement: MMC S1 — Mycelium grown in porous scaffolds results in higher biomass and mechanical properties compared to standard plate growth. [file mmc1.pdf]

Supporting Information
Mycelial Growth of Wood Fungi in Porous Scaffolds

Natalie Nussbaum,*,† Nils Repond,‡ Antoni Gandia,¶ Peter Fischer,*,† and Patrick A. Rühs†

†*Eidgenössische Technische Hochschule Zürich, Department of Health Sciences and Technology, 8092 Zürich, Switzerland*

‡*Eidgenössische Technische Hochschule Zürich, Department of Materials, 8092 Zürich, Switzerland*

¶*Polytechnic University of Valencia, Institute for Plant Molecular and Cell Biology, 46022 Valencia, Spain*

E-mail: natalie.nussbaum@hest.ethz.ch; peter.fischer@hest.ethz.ch

Gelation temperature of agar substrate

To determine the gelation temperature of the SMA substrate, a temperature sweep was conducted using a rheometer (Anton Paar MCR 702) equipped with a concentric cylinder geometry (CC17) featuring a 4.73 ml cup volume. A linear temperature ramp was applied, starting at 60°C and decreasing to 30°C, with a resolution of 1 point per °C. The test was strain-controlled to prevent the generation of excessive shear rates, which could interfere with the gel formation process. The strain γ was set at 1%, and the angular frequency (ω) was maintained at 1 rad/s. Liquid agar, heated to approximately 85°C, was transferred directly

from its heat source into the preheated rheometer cup and allowed to stabilize thermally. Once the temperature reached 60°C, the experiment was started.

Figure S2 shows the results of the temperature sweep (n=3). The gelation point, defined as the temperature at which the storage modulus G' intersects with the loss modulus G",[1] was determined to be 46.26°C.

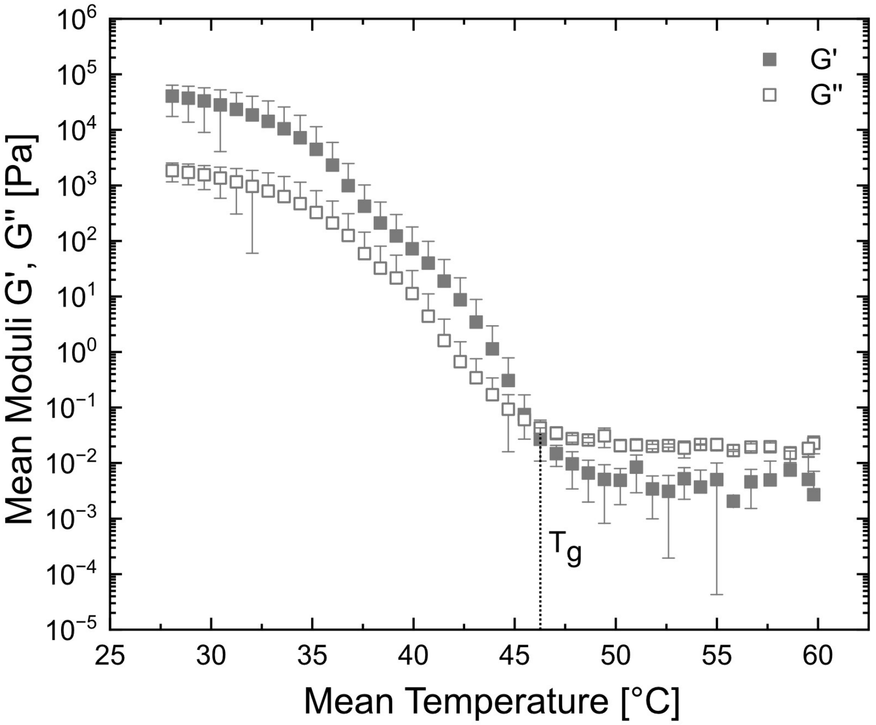

Figure S1: Temperature sweep of the standard malt agar (SMA) substrate

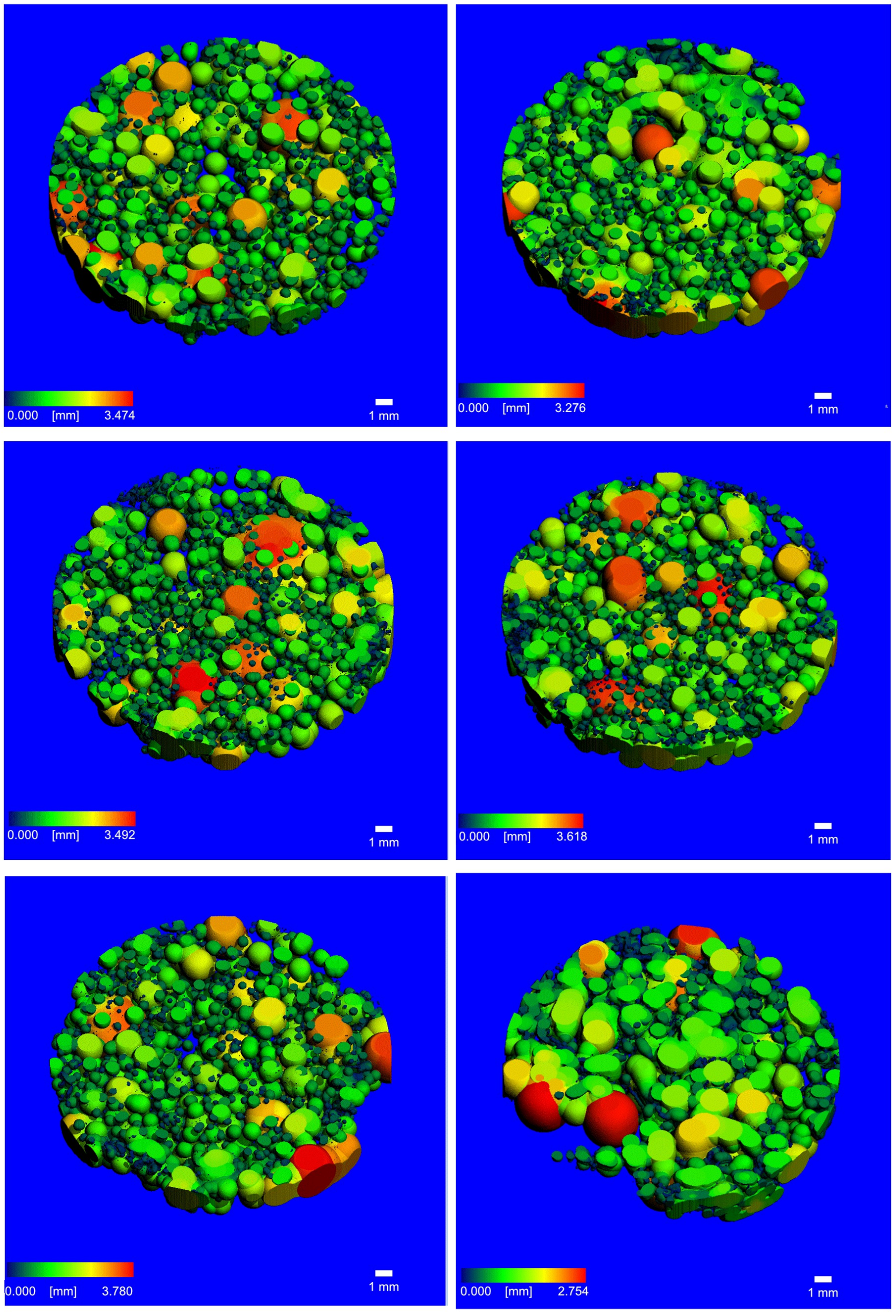

Figure S2: Micro-CT images of hydrogel scaffolds showing qualitative pore structure and distribution. Scale bars (1 mm) are included in each image. The images illustrate the general porosity and scaffold architecture.

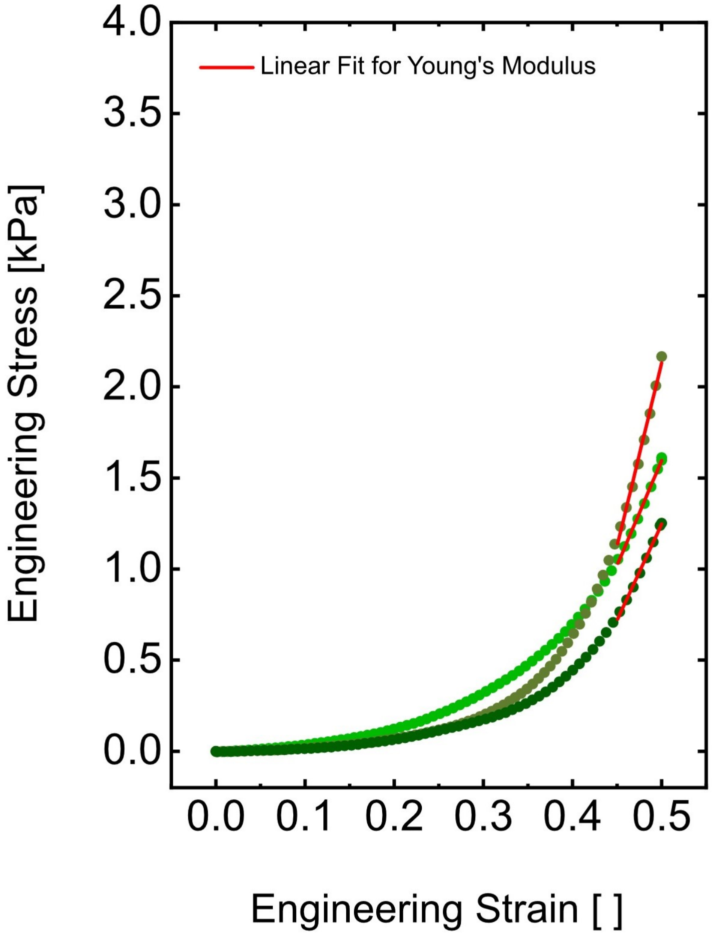

Figure S3: Representative Stress-Strain curves obtained from uniaxial tensile testing using a Zwick testing device and the fitting of the curves in the linear region.

ANOVA

Table S1: Results from two-way ANOVA test of quantified ergosterol data among various fungal strains, including degrees of freedom (DF), Sum of Squares (SS), Mean Squares (MS), F value and p value.

Substrate, Strain	DF	SS	MS	F value	p value
Interaction	2	0.658	0.329	13.280	3.990E-4

Table S2: Results of pairwise comparisons following the ANOVA from two-way ANOVA test of quantified ergosterol data among various fungal strains, including Mean difference, SEM, q value, p-value, Sig, LCL and UCL. Sig = 1 denotes statistical significance at = 0.05

Group Comparison	MeanDiff	SEM	q value	p value	Sig	LCL	UCL
GS vs GL (Plate)	-0.657	0.111	8.352	2.682E-4	1	-1.016	-0.290
GS vs PO (Plate)	-0.869	0.111	11.040	$<$ 0.0001	1	-1.228	-0.510
PO vs GL (Plate)	-0.212	0.111	2.687	0.437	0	0.570	0.147
GL vs GS (Foam)	-1.375	0.120	16.173	$<$ 0.0001	1	-1.762	-0.988
GS vs PO (Foam)	-1.595	0.120	18.761	$<$ 0.0001	1	-1.982	-1.207
PO vs GL (Foam)	-0.22	0.129	2.421	0.544	0	-0.634	0.194
Foam vs Plate (GS)	0.678	0.111	8.614	1.903E-4	1	0.319	1.037
Foam vs Plate (GL)	-0.039	0.120	0.466	0.999	0	-0.427	0.347
Foam vs Plate (PO)	-0.048	0.120	0.566	0.998	0	-0.435	0.340

Descriptive statistics

Table S3: Results from two-sample t-tests of quantified fungal biomass from growth on plate vs. scaffold substrates, including Mean values (M), t-values (t), degrees of freedom (DF) and p-values.

Sample	$M_{plate}[mg/mg]$	$M_{scaffold}[mg/mg]$	t	DF	$p-value$
2 wt% ME, 3.5 d	0.30, SEM = 0.017	0.586, SEM = 0.072	-6.59	4	0.00275
2 wt% ME, 7 d	1.2, SEM = 0.14	1.9, SEM = 0.11	-3.79		0.0091
2 wt% ME, 14 d	2.18, SEM = 0.25	3.76, SEM = 0.26	-8.77	6	1.21E-04
4 wt% ME, 7 d	0.512, SEM = 0.862	0.813, SEM = 0.159	- 2.87	4	0.04532

Table S4: Results from two-sample t-tests of quantified biomass/growth area of fungal growth on plate vs. scaffold substrates, including Mean values (M), t-values (t), degrees of freedom (DF) and p-values.

Sample	$M_{plate}[mg/mm^2]$	$M_{scaffold}[mg/mm^2]$	t	DF	$p-value$
0.5 wt% ME, 7 d	5.22 E-5, SEM = 1.95 E-6	3.58 E-5, SEM = 4.02 E-6	3.68	4	0.021
1 wt% ME, 7 d	8.46 E-5, SEM = 3.06 E-6	6.41 E-5, SEM = 8.50 E-7	5.55	5	0.0026

Table S5: Results from two-sample t-tests for the apparent stiffness (Young's modulus) of native vs. colonized scaffolds after 7 and 14 days of incubation, including Mean E- moduli (E), t-values (t), degrees of freedom (DF) and p-values.

Time of incubation [d]	$E_{native}[kPa]$	$E_{colonized}[kPa]$	t	DF	$p-value$
7	3.27, SEM = 0.60	9.82, SEM = 1.06	-5.72	12	$<$ 0.0001
14	3.22 , SEM = 0.45	15.98, SEM = 3.90	-3.25	5.13*	0.022

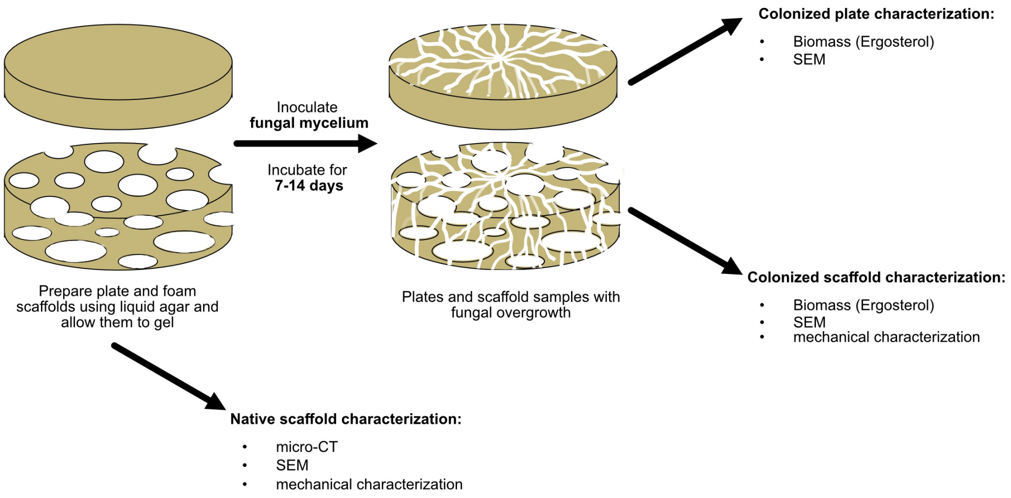

Figure S4: Workflow schematic illustrating the experimental steps and corresponding analyses performed at each stage.

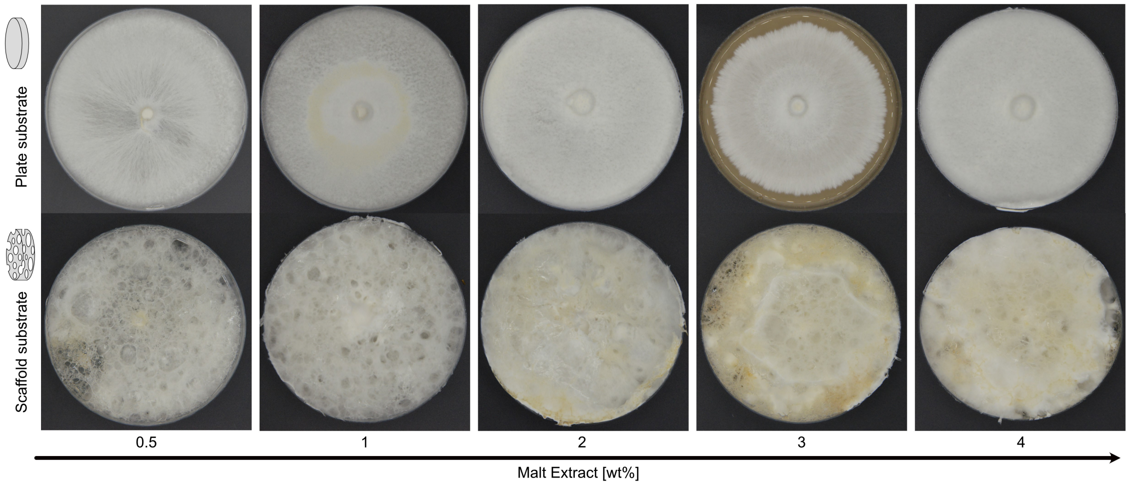

Figure S5: Morphology of *G.sessile* fungal cultures after 7 days of incubation on substrate with varying malt extract concentrations.

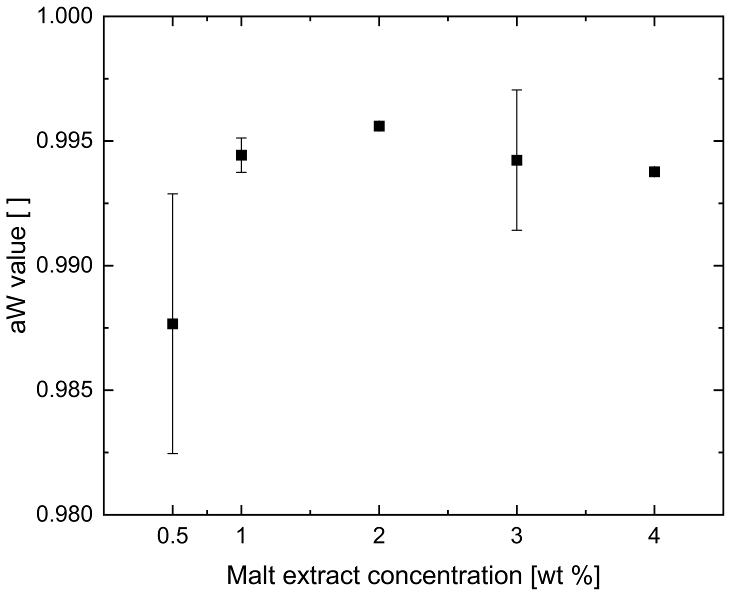

Figure S6: Water activity of solid agar substrate with varying malt extract concentrations.

Surface area scaffold vs. plate: Calculations

In this section, we offer detailed calculations of the surface area of plate or scaffold substrates, which was used to calculate the biomass in relationship to the available surface in the results section. For the plate, the area A_{plate} could easily be estimated, knowing the radius of the Petri dish r (1).

$$A_{plate} = \pi * r^2 \tag{1}$$

For the scaffold, the surface area $A_{scaffold}$ was calculated by taking the average surface area A_{MCT} obtained from the image analysis of the microtomography data (2). This average was then proportionally adjusted based on the measured sample diameter for the MCT analysis d_{MCT} and the diameter of the scaffold d_{sample} used for colonization. (which was the same for the plate and scaffold).

$$A_{scaffold} = \frac{1}{n} * \sum_{i=1}^{n} A_{MCT} * \frac{d_{sample}}{d_{MCT}} \quad (2)$$

Table S6 shows the resulting values for the respective surface areas.

Table S6: Calculation of Surface Area for Biomass Quantification

Sample	$A_{MCT}[mm^2]$	$d_{MCT}[mm]$	$d_{sample}[mm]$	Surface Area $[mm^2]$
Plate	-	-	90	6361.73
Scaffold	2353.79 ± 55.67	28	90	7779.40

References

(1) Mezger, T. G. *Vincentz Network*; 2014; pp 790–797.
